# Ameliorating effects of natural herbal supplements against water-borne induced toxicity of heavy metals on Nile tilapia, (*Oreochromis niloticus*)

**DOI:** 10.1038/s41598-024-72268-4

**Published:** 2024-09-29

**Authors:** Arzoo Fatima, Syed Makhdoom Hussain, Shafaqat Ali, Muhammad Rizwan, Khalid A. Al-Ghanim, Jean Wan Hong Yong

**Affiliations:** 1 Fish Nutrition Lab, Department of Zoology, Government College University Faisalabad, Faisalabad, Punjab 38000 Pakistan; 2 Department of Environmental Sciences, Government College University Faisalabad, Faisalabad, Punjab 38000 Pakistan; 3 Department of Biological Sciences and Technology, China Medical University, Taichung, 40402 Taiwan; 4 Department of Zoology, College of Science, King Saud University, 11451 Riyadh, Saudi Arabia; 5 Department of Biosystems and Technology, Swedish University of Agricultural Sciences, 23456 Alnarp, Sweden

**Keywords:** Heavy metal, Herbal supplement, Growth, Histopathology, Nile tilapia, Toxicity, Zoology, Environmental sciences, Natural hazards, Health care

## Abstract

The efficacy of herbal supplements in mitigating heavy metals (HMs) toxicity was investigated using a widely grown fish, the Nile tilapia (*Oreochromis niloticus*). The experiment was conducted over two phases: during the stress phase, the experimental fishes were exposed to sub-lethal concentrations of HMs, including lead, cadmium, zinc, and copper for 15 days; following which during the feeding phase, herbal supplements were given for 70 days to ameliorate their effects. Seven groups were established: the control negative group (CON−ve), control positive group (CON+ve, without any treatment), and five groups with supplementation of 1% turmeric (TUR), cinnamon (CIN), ginger (GIN), garlic (GAR), and their mixture (MIX), respectively. A total of 315 fishes were distributed evenly in experimental tanks (15 fishes per tank, in triplicates). The results revealed that exposure to HMs led to significant (*p* < 0.05) alterations in all the tested parameters, i.e., liver damage and growth reduction. The herbal supplements, especially the MIX groups, ameliorated the harmful effects of HMs and restored fish growth, digestibility, carcass composition, and liver health. In conclusion, the study demonstrated that the herbal supplements were effective in reducing the HMs-linked toxicity in Nile tilapia. Future studies pertaining to the mechanisms facilitated by the various herbal bioactive substances-linked tolerance to HMs in fishes are warranted.

## Introduction

Aquaculture, the farming of marine and aquatic organisms, is a significant and fastest food-producing sector in the world. As the traditional capture fisheries are stagnating, associated with the general weakening of the global marine food webs, aquaculture is filling the deficit in providing food to a growing world population^1,2^. Heavy metals (HMs) pollution has emerged as a serious issue among other pollutants in the aquacultural sector due to their low density < 5 g/cm^3^, potential toxicity, non-biodegradable nature, tendency to bio-accumulate in tissues, and extended half-life^3,4–6^. These metals (heavy metals and metalloids) enter the aquatic habitats from various natural and anthropogenic sources, such as domestic waste, pollution, industrial effluents and atmospheric emissions. HMs accumulate in aquatic organisms and cause various ailments when certain biological thresholds are exceeded^5–7, 8^. As fishes form part of the human diet, the consumption of fishes with high levels of tissue HMs will eventually cause serious health issues for these individuals. It is well known that the introduction of HMs into aquatic ecosystems and water bodies has a significant impact on the entire food chain. Additionally, HMs have greater influence on the various ecosystems because they persist for prolonged periods that ultimately lower the quality of the water^5,10^.

 Good nutrition and the aquatic environment are the two pivotal factors to supporting the growth, development and reproduction of fish. In the process of growth in an aquatic environment, fish species in this industry are exposed to HMs in a variety of ways, such as by ingesting, taking through the gills, consuming inedible particles, and absorption through the skin^11^. These metals accumulate in fish organs such as liver, gills and kidneys. Their excessive accumulation will cause many physiological, biochemical and histological changes in aquaculture species. The presence of HMs in aquaculture is influenced by the size of the fish, exposure duration of HMs, route of exposure of HMs, amount of HMs and experimental settings^5, 6,10^. Recently, Giri et al.^9^ found that when *Cyprinus carpio* was exposed to Pb, it exhibited growth suppression, histopathological and biochemical alterations and oxidative stress.

In the past, scientists often controlled pathogens or infections using chemicals and antibiotics. Although these biological and chemical agents have detrimental impacts on the whole ecosystem when utilized in high dosage and over a long term basis^2,6,12^. Additionally, antibiotics develop resistance, inhibit the immune system, and accumulate residues in fish organs^13^. Therefore, it is crucial to enhance the understanding of complementary medicines that can effectively control fish diseases in the long run. Recent studies found that the impacts of medicinal plants on health of fishes have become more advantageous as a result of the growing economic and environmental relevance, since they may help develop disease prevention and control measures. These plants can act as growth promoters, antistress agents, or immunostimulants^12^.

Using natural supplements in aquaculture production is seen to be an effective measure to strengthen fish immunity and also creating a healthy aquatic environment^14– 16^. Specifically, various botanicals and including Indian and Chinese herbal medicines may serve as successful supplements to improve fish growth, development and health by formulating special fishmeal with selected supplements and including immunostimulants^15,17, 18–24^. These reported efficacies are due to the abundance of bioactive components, including flavonoids, polysaccharides, saponins, polyphenols, essential oils, terpenoids and alkaloids^25–31^. These substances have been utilized extensively in improving growth, organ and tissue function, nutrient metabolism, controlling bacterial and viral infections^14,16,21,25^. Garlic, a medicinal herb, has a variety of impacts on fishes. In addition to having antibacterial and antifungal qualities, it also has beneficial effects on flesh quality, immunological function, body metabolism, and growth^1^. Natural antioxidants like flavonoids are also present in garlic^32^. It has some bioactive components that enhance fish resistance to diseases in aquaculture^33^. Another herb, turmeric has been used extensively in both human and animal medicine^34^. In aquaculture, turmeric and its active ingredients have a variety of therapeutic characteristics, including anti-inflammatory, immunomodulatory, anti-stress, antibacterial and hepatoprotective properties^35^. Similarly, in tropical areas of the world, cinnamon (*Cinnamomum* sp.) is a highly prized and widely used spice with immunostimulant and antioxidant characteristics^36^. The cinnamon contains bioactive elements i.e., phenolic compounds, minerals, essential oils, and vitamins^37^. It is also utilised in aquaculture as a nutritional feed additive to strengthen the immune systems in fishes^36^. Ginger, a palatable herb, also known as *Zingiber offcinale*, has also been used effectively in aquatic species to enhance growth, immunity, and tolerance to several harmful bacteria and parasites^17^. Due to the strong antibacterial effects delivered by ginger, several aquatic animals with bacterial infections have shown remarkable resistance to infection^18^.

Nile tilapia holds the position of the world's second most cultivated species, after the carp^38,39^. This is considered to be a promising species for general aquacultural production worldwide^40^. Globally, the tilapia production has experienced a remarkable surge, expanding from a modest 0.5 million metric tonnes (MMT) in the early 1990s to a substantial 6.03 MMT in 2018, representing an average annual growth rate of 13.5%^39^. Based on many reports, the Nile tilapia has proven to be an ideal species for the global aquaculture system due to its good tolerance to poor water conditions and general zoonotic diseases. It has a relatively fast growth rate and is considered the best consumer choice of fish in many societies^41^. The detailed study examined the potential alternative strategy of using commonly available herbal materials to alleviate HM-induced stress on the Nile tilapia. This research specifically determined the ameliorating effects of four natural herbal supplements (garlic, turmeric, cinnamon, ginger) against the water-borne linked toxicity of HMs on tilapia.

## Materials and methods

### Ethical statement

This study was executed in strict accordance with the ethical guidelines and protocols approved by the Animal Welfare and Ethics Committee of Government College University Faisalabad, adhering to the ARRIVE guidelines (Ref No. GCUF/ERC/443).

### Experimental conditions and fishes

Fishes were procured from the local hatchery and taken to the Government Fish Nutrition Laboratory and placed for two weeks in V-shaped steel tanks for acclimatization. During this time period, they were given basal diet (SFM). To prevent infections, the fishes were treated with a solution containing 5% NaCl^42^. To provide a conducive environment for fish growth and development, the capillary method was utilized for providing aeration and optimal water quality parameters were maintained constantly (pH: 7.4–8.5, temperature: 24.8–28.6 °C and dissolved oxygen: 4–6 mg/L g).

### Preparation of heavy metals mixture

The HM salts (100 µg/L of lead acetate/Pb (C_2_H_3_O_2_)_2_·3H_2_O; 2.5 mg/L of copper sulfate/CuSO_4_·5H_2_O; 800 mg/L of zinc sulfate/ZnSO_4_·7H_2_O and 250 of µg/L cadmium chloride/CdCl_2_) were obtained from the Biochemistry Laboratory, Government College University Faisalabad. To make a stock solution, these compounds were first dissolved in distilled water (1000 mL) and the required concentrations were prepared by diluting the stock solution^43,44^.

### Experimental design

The experiments were carried out in two phases.

### Phase 1 (stress phase)

Following a 15-day stress period, the acclimatized *O. niloticus* (bulk weight: 925 g) were transferred to aquaria containing 70 L of water. These fish were distributed evenly, with 16 fish per aquarium, and held there for two weeks. Afterwards, the following groups were maintained: first negative group of HMs in which the fishes were fed on a basal diet (SFM) and placed in HMs free water; the second positive group of HMs in which the fishes were subjected to an exposure of HMs mixture (sub lethal dosage of all metals) and fed with a basic diet. The maintenance of HMs at a constant level was achieved by changing the solutions after every two days with water containing HMs, in the HMs treated group. In control groups, 20% of water was changed on daily basis and 100% water was changed in a week^9^.

### Phase II (feeding phase)

All fish, excluding those in the control group, were randomly relocated to V-shaped tanks with specialized valves, and were fed diets enriched with herbal supplements for 70 days. In the feeding phase, total of 315 fingerlings were distributed into experimental tanks, 15 fingerlings per tank in triplicates. The experimental design consisted of seven treatment groups. The first and second groups were the control negative (CON−ve) and positive (CON+ve), respectively, which had no herbal supplement. The remaining groups (3rd to 7th group) contains 1% of each herbal supplements i.e turmeric (TUR), ginger (GIN), cinnamon (CIN), garlic (GAR) and the combination of all herbs (MIX), respectively^19^.

### Preparation of feed and experimental diets

Herbal supplements (TUR, GIN, CIN, GAR) and all other ingredients were taken from local market, Faisalabad (Table 1) and their proximate was tested using the standard protocols^45^. Then they were crushed and mixed homogeneously. After that, water and fish oil was added to form dough. Seven SFM-based experimental diets were prepared out of which control groups (CON−ve and CON+ve) without herbal supplements and other five groups had 1% herbal supplements. Prepared pellets were stored in oven at 105 °C for 24 h^46^.

**Table 1:** The composition of the ingredients used in the experimental diets for the fishes.

Ingredients	Control (CON−ve / CON+ve )	TUR (turmeric)	CIN (cinnamon)	GIN (ginger)	GAR (garlic)	MIX (mixture)
Herbal supplements (%)	0	1	1	1	1	1
Sunflower meal	52	52	52	52	52	52
Wheat flour*	12	11	11	11	11	11
Fish meal	16	16	16	16	16	16
Rice Polish	9	9	9	9	9	9
Chromic oxide	1	1	1	1	1	1
Fish oil	7	7	7	7	7	7
Mineral premix**	1	1	1	1	1	1
Ascorbic acid	1	1	1	1	1	1
Vitamin Premix***	1	1	1	1	1	1

*Wheat flour was substituted with the herbal supplements.

**Mineral premix kg^−1^: Fe: 9500 mg, Co: 41 mg, Cu: 590 mg, P: 136 g, Ca: 150 g, Se: 3 mg, Mn: 2100 mg, Na: 43 g, Zn: 3200 mg, Mg: 56 g, I: 42 mg.

***Vitamin (Vit.) premix kg^−1^: Vit. B12: 40 mg, Vit. A: 14,000,000 IU, Nicotinic acid: 61,000 mg, Vit. C: 13,000 mg, B2: 8000 mg, Vit. B6: 3500 mg, Vit. Ca pantothenate: 12,500 mg, Vit. D3: 3,000,000 IU, Folic acid: 1400 mg, Vit. K3: 7000 mg.

### Feeding protocol and sampling

During the feeding phase, the fishes were fed twice daily at a rate equivalent to 5% wet weight (morning and afternoon). Following the 2-hour feeding period, the excess diet was drained from through the valves. The food debris in the tanks were carefully cleaned before being replaced with water. Specifically, the collection of faeces was done by opening the valves, in a similar way. Care was required not to break the delicate fecal threads in order to reduce the estimation of nutrient loss^47^.

### Growth assessment

The growth were calculated by using a standard formulae^47^.

$$\text{WG }(\text{g})= (\text{Final weight}-\text{Initial weight})$$

$$\text{SGR }= (\text{In }(\text{Final weight}) -\text{In }(\text{Initial weight}) \times 100 /\text{ No}.\text{ of days}$$

$$\text{WG\%}= (\text{Final weight}-\text{Initial weight})\times 100 /\text{ Initial weight}$$

$$\text{FCR }=\text{ Total dry feed intake }(\text{g}) /\text{ Wet weight gain}$$

### Proximate analysis

After a 70-day of feeding phase, four fingerlings from each group were chosen for analysis of carcass, while 1 g of feed and faeces samples from every tank were collected for digestibility assessment. The samples were then homogenized and the findings were analyzed according to established protocols^45^. By using the micro Kjeldahl apparatus, crude protein (CP) in faeces and diet was studied. Oxygen bomb calorimeter was utilized to determined gross energy (GE). The Soxhlet HT2 1045 apparatus, utilizing petroleum ether, was employed to extract and determine the crude fat (CF) content.

### Hematology study

Blood was taken from caudal blood vessels for the analysis of hematological indices. Three fish samples from each tank were taken after giving them solution of clove oil. Hematocrit or packed cell volume (PCV) calculation was taken by using micro-hematocrit by using capillary tube. Calculation of white blood cells (WBCs), platelets (PLTs) and red blood (RBCs) cells was determined by Neubauer chamber^48^. By using method of Wedemeyer and Yasutake^49^, hemoglobin (Hb) were measured. To determine the mean corpuscular volume (MCV), mean corpuscular hemoglobin (MCH) and corpuscular hemoglobin concentration (MCHC) following formulae were used by following^47^.

$$\text{MCV }=\text{ PCV }/\text{ RBC }\times 10$$

$$\text{MCHC }=\text{ Hb }/\text{ PCV }\times 100$$

$$\text{MCH }=\text{ Hb }/\text{ RBC }\times 10$$

### Digestibility

Using a standardized formula^47,50^, the digestibility (ADC%) was computed for the feed and fecal samples.

### Histopathology of liver

One fish from each tank was selected for dissection, and its liver was extracted. After that, the livers of fingerlings were then immersed in 10% neutral buffered formalin, where they were fixed for 24 hours. The tissue were dehydrated in ethanol at varying levels before being set in paraffin wax. Hematoxylin and eosin were used to stain sagittal sections that were 6 mm thick before being processed for light microscopy^43,51^.

### Statistical analysis

Using ANOVA and Tukey’s Honesty Significant (HSD) Test, the data of fingerlings were analyzed and differences in groups were compared^52,53^ *p* < 0.05 was considered a significant difference. 

## Results

### Growth assessment

**Fig. 1:**
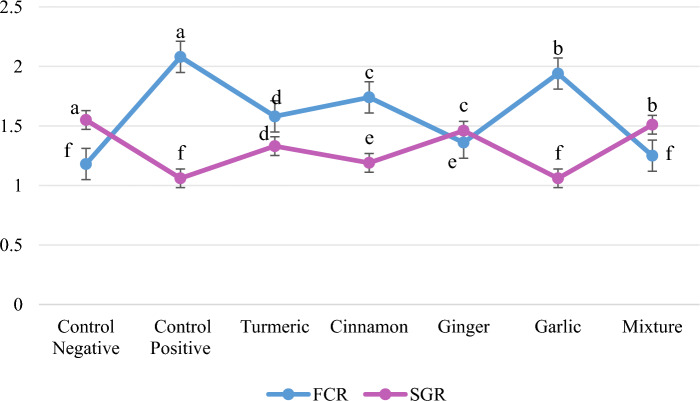
The FCR and SGR% of *O. niloticus* fed natural herbal supplemented diets.

The growth indices of *O. niloticus* fed on SFM based experimental diets having graded levels (0%, and 1% of TUR, CIN, GIN, GAR and MIX) is represented in Table 2. When compared with CON+ve diet, natural herbal supplements considerably improve growth performance of *O. niloticus.* The maximum weight gain (g) (17.37 ± 0.12) and the least FCR (1.18 ± 0.03) was recorded in CON−ve group while the second best results were observed in MIX group which was substantially different (*p* < 0.05) from all experimental levels. However, minimum weight gain (9.74 ± 0.20) and highest FCR (2.08 ± 0.05) were noticed in CON+ve (without any herbal supplement) (Fig. 1).

**Table 2:** Growth indices of *O. niloticus* fed natural herbal supplemented diets. The presented data are means of three replicates.

Experimental diets	Natural herbal supplements (%)	Initial weight (g)	Final weight (g)	Weight gain (%)	Weight gain(g)
CON−ve	0	8.83 ± 0.02^ab^	26.20 ± 0.11^a^	196.76 ± 1.37^a^	17.37 ± 0.12^a^
CON+ve	0	8.82 ± 0.01^b^	18.56 ± 0.02^f^	110.50 ± 2.31^f^	9.74 ± 0.20^f^
TUR (turmeric)	1	8.83 ± 0.01^ab^	22.45 ± 0.10^d^	154.32 ± 0.99^d^	13.62 ± 0.09^d^
CIN (cinnamon)	1	8.85 ± 0.01^ab^	20.32 ± 0.06^e^	129.51 ± 0.99^e^	11.46 ± 0.07^e^
GIN (ginger)	1	8.85 ± 0.01^ab^	24.62 ± 0.06^c^	178.22 ± 0.52^c^	15.77 ± 0.05^c^
GAR (garlic)	1	8.86 ± 0.01^a^	18.63 ± 0.15^f^	110.30 ± 1.49^f^	9.77 ± 0.14^f^
MIX (mixture)	1	8.84 ± 0.02^ab^	25.53 ± 0.07^b^	188.66 ± 1.29^b^	16.69 ± 0.08^b^

^a–f^Means having different superscripts are substantially different.

### Body composition

**Fig. 2:**
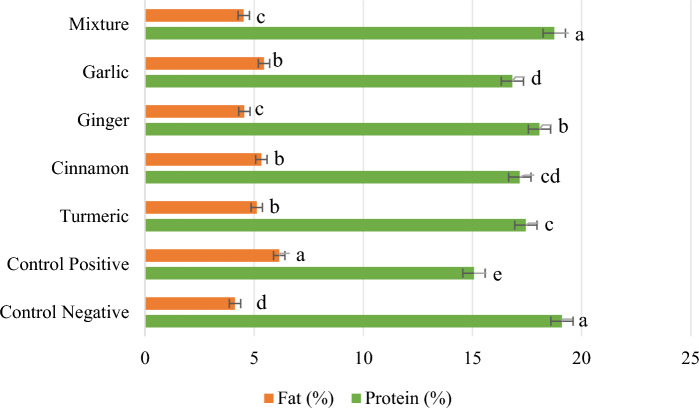
Graphical representation of body composition in terms of crude protein (%) and crude fat (%).

The impacts of herbal supplemented diets on carcass of *O. niloticus* are shown in Table 3. When fish were fed SFM diet supplemented with herbal supplements, the results showed the highest crude protein (19.10 ± 0.09%) and best crude fat (4.12 ± 0.11%) at CON−ve group (without HMs exposure). However, a significant improvement (*p* < *0.05*) in body composition was noted in tilapia when fed with herbal supplements, compared to the CON+ve group. Although, the MIX group exhibited the second-best body composition outcomes, whereas the CON+ve group showed the poorest results, with significantly lower protein content (15.07 ± 0.29%) and higher fat content (6.15 ± 0.14%) (Fig. 2). In terms of moisture and ash, all the experimental diets showed an almost non-significant impact when compared to CON groups.

**Table 3:** Ash and moisture content of *O. niloticus* fed on natural herbal supplements. The data presented are means of three replicates.

Experimental diets	Natural herbal supplements (%)	Ash (%)	Moisture (%)
CON−ve	0	1.27 ± 0.12^c^	75.50 ± 0.22^bc^
CON+ve	0	2.59 ± 0.25^a^	76.18 ± 0.17^a^
TUR (turmeric)	1	1.76 ± 0.09^b^	75.65 ± 0.11^b^
CIN (cinnamon)	1	1.79 ± 0.04^b^	75.69 ± 0.10^b^
GIN (ginger)	1	1.74 ± 0.10^b^	75.63 ± 0.07^b^
GAR (garlic)	1	1.83 ± 0.11^b^	75.88 ± 0.09^ab^
MIX (mixture)	1	1.54 ± 0.13^bc^	75.17 ± 0.16^c^

^a–c^Means having different superscripts are substantially different.

### Hematological indices

The outcomes of blood parameters of *O. niloticus* are shown in the Table 4. The best results of hematological parameters were seen in CON−ve group. In comparison to other diets, MIX group had the second highest values of hematological indices in Nile tilapia. There is a notable (*p* < 0.05) increase in RBCs (4.43 ± 0.08 × 10^6^ mm^−3^), WBCs (9.27 ± 0.12 × 10^3^ mm^−3^), Hb (8.75 ± 0.06 g/100 mL), PLTs (60.11 ± 0.12) and other blood parameters at CON−ve group that are not exposed to HMs. Whereas the least hematological indices were observed at CON+ve group when compared to other supplemented diets.

**Table 4:** Hematological parameters of *O. niloticus* fed natural herbal supplements based diets. The data presented are means of three replicates.

Experimental diets	Natural herbal supplements (%)	RBCs (10^6^ mm^−3^)	Hb (g/100 mL)	WBCs (10^3^ mm^−3^)	PCV (%)	PLT	MCHC (%)	MCH (fl)	MCV (pg)
CON−ve	0	4.43 ± 0.08^a^	8.75 ± 0.06^a^	9.27 ± 0.12^a^	22.93 ± 0.05^ab^	60.11 ± 0.12^a^	38.44 ± 0.05^a^	58.26 ± 0.09^b^	195.97 ± 3.66^a^
CON+ve	0	2.34 ± 0.13^f^	5.25 ± 0.14^f^	7.14 ± 0.03^f^	20.94 ± 0.03^b^	57.65 ± 0.04^f^	34.06 ± 0.02^ g^	72.63 ± 0.06^a^	96.81 ± 4.04^f^
TUR (turmeric)	1	3.12 ± 0.03^d^	7.84 ± 0.03^c^	8.64 ± 0.01^c^	23.47 ± 2.33^a^	58.96 ± 0.03^c^	37.81 ± 0.05^d^	55.83 ± 0.04^e^	184.05 ± 2.16^c^
CIN (cinnamon)	1	2.90 ± 0.90^de^	7.45 ± 0.02^d^	7.92 ± 0.04^d^	21.94 ± 0.03^ab^	58.74 ± 0.4^d^	36.94 ± 0.04^e^	54.84 ± 0.04^f^	174.67 ± 3.04^d^
GIN (ginger)	1	3.55 ± 0.04^c^	8.48 ± 0.03^b^	8.94 ± 0.02^b^	22.58 ± 0.04^ab^	59.12 ± 0.03^b^	38.03 ± 0.03^c^	56.92 ± 0.03^d^	185.56 ± 1.61^bc^
GAR (garlic)	1	2.76 ± 0.02^e^	7.03 ± 0.04^e^	7.48 ± 0.02^e^	21.01 ± 0.03^b^	58.01 ± 0.03^e^	35.53 ± 0.03^f^	53.81 ± 0.04^ g^	164.45 ± 1.36^e^
MIX (mixture)	1	4.23 ± 0.02^b^	8.57 ± 0.05^ab^	9.06 ± 0.02^b^	22.81 ± 0.03^ab^	59.92 ± 0.03^a^	38.22 ± 0.03^b^	57.82 ± 0.05^c^	192.29 ± 3.28^ab^

*RBC* red blood cell, *Hb* hemoglobin, *WBC* white blood cell, *PCV* packed cell volume, *PLT* platelet, *MCV* mean corpuscular volume, MCHC mean corpuscular hemoglobin concentration, *MCH* mean corpuscular hemoglobin.

^a–g^Means having different superscripts are substantially different.

### Nutrient digestibility

**Fig. 3:**
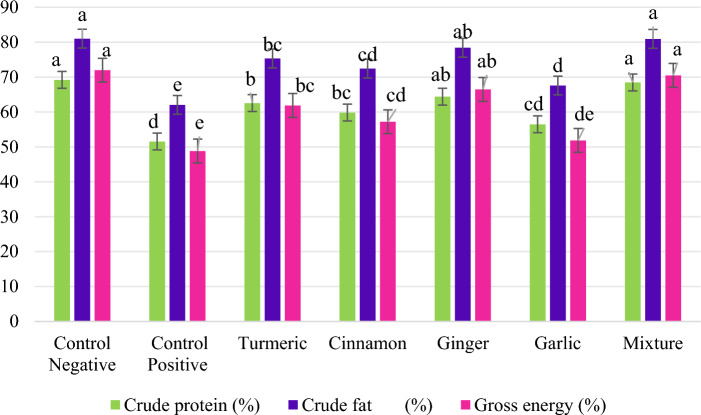
The apparent nutrient digestibility (%) of *O. niloticus* fed with natural herbal supplemented diets.

Tables 5, 6 and Fig. 3 show the nutrient analysis in feed, faeces and ADC% when fed with SFM diet supplemented with herbal supplements. Fish fed with natural herbal supplements in their feed had the minimum amount of nutrients loss in their faeces. Thus, the least loss of nutrients in faeces was noted in control negative group (Table 6). However, the analyzed composition of feed in all experimental diets were non-significant (*p* > 0.05) to each other (Table 5). In terms of ADC%, the best CP (69.20 ± 3.23%), CF (81.01 ± 1.29%) and GE (71.99 ± 1.95%) were recorded in the CON−ve group, followed by the MIX group.

**Table 5:** The analyzed digestibility of apparent crude protein (CP; %), gross energy (GE; %) and crude fat (CF; %) in feed of *O. niloticus* fed on natural herbal supplemented SFM based diet.

Test diets	Natural herbal supplement (%)	CP (%)	CF (%)	GE (kcal g^−1^)
CON−ve	0	30.11 ± 0.07	8.13 ± 0.03	3.56 ± 0.03
CON+ve	0	30.13 ± 0.05	8.12 ± 0.02	3.57 ± 0.02
TUR (turmeric)	1	30.13 ± 0.08	8.15 ± 0.03	3.55 ± 0.06
CIN (cinnamon)	1	30.11 ± 0.06	8.14 ± 0.03	3.56 ± 0.02
GIN (ginger)	1	30.14 ± 0.05	8.14 ± 0.04	3.58 ± 0.02
GAR (garlic)	1	30.10 ± 0.08	8.14 ± 0.03	3.57 ± 0.03
MIX (mixture)	1	30.12 ± 0.07	8.14 ± 0.04	3.57 ± 0.02

The data presented are means of three replicates.

*CF* crude fat, *CP* crude protein, and *GE* gross energy.

**Table 6:** The analyzed digestibility (%) of faeces of *O. niloticus* fed on natural herbal supplemented SFM based diet

Test diets	Natural herbal supplements	CP (%)	CF (%)	GE (kcal g^−1^)
CON−ve	0	11.35 ± 0.57^d^	1.83 ± 0.07^e^	1.20 ± 0.07^e^
CON+ve	0	17.58 ± 0.28^a^	3.71 ± 0.18^a^	2.20 ± 0.07^a^
TUR (turmeric)	1	13.62 ± 0.21^c^	2.34 ± 0.13^d^	1.63 ± 0.09^c^
CIN (cinnamon)	1	14.68 ± 0.26^b^	2.72 ± 0.16^c^	1.85 ± 0.08^b^
GIN (ginger)	1	12.80 ± 0.32^c^	2.09 ± 0.12^de^	1.43 ± 0.08^cd^
GAR (garlic)	1	15.61 ± 0.26^b^	3.14 ± 0.09^b^	2.05 ± 0.07^ab^
MIX (mixture)	1	11.44 ± 0.52^d^	1.87 ± 0.09^e^	1.27 ± 0.08^de^

The data presented are the means of three replicates.

^a–e^Means having different superscripts are substantially different.

*CF* crude fat, *CP* crude protein, and *GE* gross energy.

### Liver histopathological findings

**Fig. 4:**
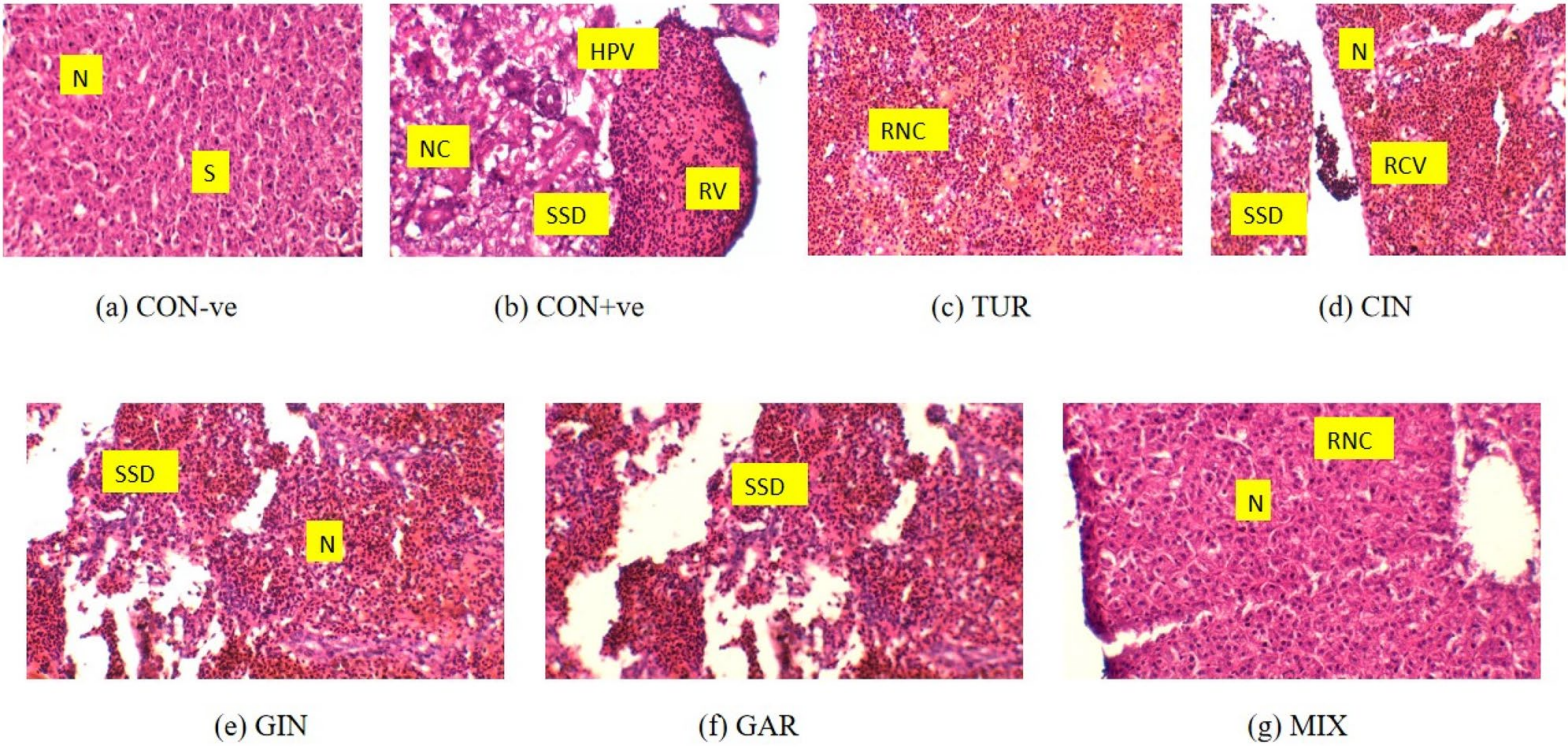
Representative photomicrographs of liver tissues from the different groups of *O. niloticus* fed with heavy metals (HMs) mixture and natural herbal supplements: (**a**) Control (CON)-ve group: Normal liver histology with round nuclei (N) and intact sinusoids (S). (**b**) CON+ve group: Severe alterations, including necrosis (NC), oedema (OE), hepatocellular vacuolation (HPV), pyknosis (PC), and sinusoid dilation (SD). (**c**) Turmeric (TUR)-treated group: Improved liver histology with reduced NC. (**d**) Cinnamon (CIN)-treated group: Restored normal liver architecture, with round N, recovered central vein damage (CV) and stopped sinusoid dilation (SD). (**e**) Ginger (GIN)-treated group: Normalized liver histology, with stopped SD and recovering round N. (**f**) Garlic (GAR)-treated group: Improved liver histology, with stopped SD. (**g**) Mixed herbal supplement (MIX)-treated group: Enhanced liver recovery, with reduced NC and normalized round N of hepatocytes (H).

Figure 4 depicts the morphology of the livers of control and treated fish. A microscopic observation of the CON−ve liver revealed a typical structure with hepatocytes, a vascular network consisting of hepatic portal veins that bring venous blood from the intestines into the liver, which then splits into capillaries known as sinusoids. The CON+ve group represents central vein damage, sinusoid dilation, swelling of hepatocellular vacuolation, odema, necrosis and pycknotic nuclei in several cells. HMs exert detrimental effects on liver function by triggering histopathological changes, which result from the generation of oxidative stress and disruption of the antioxidant defense system. Liver histopathology of *O. niloticus* fed with herbal supplements shows reduction in further sinusoidal dilation, recovery of normal round nuclei, reduction in necrosis and recovery of central vein damage.

## Discussion

Due to the flow of anthropogenic wastes into the aquatic environment, HMs damage the various natural ecosystems and food production systems. Studies indicated that aquatic animals exposed to HM toxicity hinder their growth performance and induce number of physiological problems^5,6,9,10,^. This study aimed to investigate the potential benefits of herbal supplements against the HMs exposure. The outcomes of the present study showed that adding natural herbal additives in SFM based diets improved morphological and hematological parameters of *O. niloticus*. Seven SFM based diets including 0% CON−ve and CON+ve and 1% natural herbal supplements were used to mitigate the effects of HMs and improve their growth performance, carcass composition, nutrient digestibility and hematological parameters.

In this study, the best results were shown in CON−ve group followed by MIX group of *O. niloticus*. Maximum values of weight gain (17.4 g), SGR (1.6%) and lower FCR (1.2) were noted in CON−ve group of SFM based diet. Compared with the results, the second best results were noted in MIX group. Consistent with our findings, Giri et al.^9^ reported that CUR supplementation promoted the growth of *C. carpio* and alleviated the toxicity of Pb. These growth improvements were due to the bioactive compounds (allicin, alliin, flavonoids, carotenoids), anti-inflammatory responses, growth promoting factors, immunity boosting ability, essential amino acids, vitamins and minerals in all these herbal supplements, which increased digestion and feed intake and ultimately boost fish health and well-being^33,16, 17, 35, 37^. Chowdhury et al.^19^ used the combination of GIN, GAR and TUR and found that the growth of *Labeo rohita* fingerlings improved significantly. Similar results were found by Jahanjoo et al.^54^ in Sobaity, sea bream that fed fish with 1% GIN, 1% GAR, 1% thyme and MIX of these three medicinal herbs, resulted in highest value of SGR, WG, and FCR in MIX group. Furthermore, Yousaf et al.^55^ reported that dietary CIN exhibited that *Catla catla* showed recuperative effects on growth following sub-lethal exposure to Pb.

The outcomes of carcass composition of *O. niloticus* indicated substantial improvement in MIX group when fed with natural herbal supplemented diet. Our findings are in line with Yousaf et al.^55^ who demonstrated that the carcass composition of *C. catla* improved when fed with the CIN herb along with waterborne Pb exposure. Moreover, because CIN stimulates GI secretion, it may enhance nutritional absorption and digestion, improving the quality of fish carcasses. Mohammadi et al.^20^ reported higher protein content while lower lipid, ash and moisture content in *C. carpio* were measured when fed with the GIN extracts. According to the results of Eissa et al.^56^, CP increased and ash were decreased in Nile tilapia when supplemented with nanocurcumin. Results reported by Wahyudi et al.^57^ indicated that when feeding catfish with CIN, the tissue protein content increased by 25.6 to 25.9%; associated with lower lipids, moisture and ash content in catfish. Adineh et al.^58^ evaluated that the carcass composition of rainbow trout significantly improved; with concomitant higher protein levels and lower lipid levels as compared to the CON group when GAR supplemented feed given to the fishes.

The hemato-biochemical condition of fish was significantly changed by HMs contamination and many abnormalities in different blood cells were indicated by Islam et al.^59^. In the current study, hematological parameters such as RBCs, WBCs, Hb and other hematological indices were enhanced significantly in Nile tilapia when fed with natural herbal supplemented SFM based diet. At the CON+ve group, lowest numbers of these parameters were observed. According to Giri et al.^9^, dietary CUR supplementation in common carp effectively mitigated the Pb-induced hemato-toxicity. Jahanjoo et al.^54^ found that there was significant increase in WBCs and RBCs when the fishes were fed with three medicinal herbs (GIN, GAR and thyme). Recently, Yousaf et al.^55^ reported that CIN supplementsmitigated the Pb-induced toxicity in *C. catla*. Our results also showed that the second best results of hematological markers were obtained in the MIX group in which the RBCs and WBCs showed improvements (*p* < 0.05).

The nutritional digestibility of *O. niloticus* was evaluated by faeces and feed analysis. In *O. niloticus*, the lowest values for CF digestibility (81.0 ± 2.7%), CP digestibility (69.2 ± 1.3%) and GE (72.0 ± 0.9%) were observed in CON−ve group. The findings of Zare et al.^60^ showed that GAR powder supplementation enhanced lipid and protein digestibility in Eurasian perch. The bioactive compounds in herbal supplements (organosulfur compounds in GAR, phenolic compounds in GIN, phytochemicals in TUR, flavonoids in cinnamon) could serve plausibly as the ameliorative agent for the fingerlings exposed to HMs; associated with better digestibility of the nutrients^14,5, 16^.

The liver histology of Nile tilapia was examined by sectioning of the liver and observing salient features under a light microscope. Our findings indicated that the exposures to HMs caused liver damages in the experimental fishes. Similar to our study, Indian lotus leaf powder mitigated the toxic effects of Pb, Zn, Cd and Hg and improved the negative histopathological alterations in Nile tilapia^43^. Giri et al.^9^ used CUR in *C. carpio* to reduce the accumulation of Pb in tissues. Wahyudi et al.^57^ reported that when CIN powder given to striped catfish *Pangasianodon hypophthalmus*, the fat reduction and improved histological condition of the fish livers were observed. According to our results, natural herbal supplements also improved the liver health. The best improvement in liver histology was seen in the MIX group among the herbal supplemented diets. According to Brum et al.^61^, Nile tilapia when fed GIN extract supplementation in the diet, improved the tissue damage caused by infection, by maintaining tissue shape and function. Abdelmagid et al.^62^ evaluated that GIN ameliorated the liver and gills tissues in Nile tilapia. Soror et al.^63^ findings demonstrated that GIN with other feed additives gave the best results and repaired the hepatocytes in Nile tilapia. Mosbah et al.^64^ explored the protective effects of GAR against the Cd induced toxicity in the liver of sea bass. Another study by Liu et al.^65^ also described the positive effect of incoporating a mixture of Fu-ling and GAR supplementary diet on the liver of grass carp during Pb stress; plausibly by enhancing the fish liver antioxidant status.

## Conclusion

In summary, our findings indicated that herbal supplements delivered promising efficacy in mitigating the harmful effects of HMs toxicity in Nile tilapia. The exposure of the fishes to HMs was found to have negative effects on the fish health, including liver damage, poor digestibility, meat quality and lesser growth. Specifically, the CON−ve group without HMs exposure, showed best outcomes in terms of growth, digestibility, carcass, hematology and histology; whereas the MIX group proved to be the best among the herbal supplemented treatments. Moving forward, researchers and farmers could consider using herbal supplements to strengthen the fish health and vitality to better adapt to aquatic pollutants like HMs. Future studies pertaining to the mechanisms facilitated by the various herbal bioactive substances-linked tolerance to HMs in fishes are warranted.

## Data Availability

The data that support the findings of this study are available from the corresponding author upon reasonable request.
